# Care seeking and attitudes towards treatment compliance by newly enrolled tuberculosis patients in the district treatment programme in rural western Kenya: a qualitative study

**DOI:** 10.1186/1471-2458-11-515

**Published:** 2011-06-29

**Authors:** John G Ayisi, Anna H van't Hoog, Janet A Agaya, Walter Mchembere, Peter O Nyamthimba, Odylia Muhenje, Barbara J Marston

**Affiliations:** 1 Centre for Global Health Research, Kenya Medical Research Institute, Kisumu-Maseno Rd, Kisian, P.O. Box 1578, Kisumu - 40100, Kenya; 2 Directorate of Research Management and Development, Ministry of Higher Education, Science and Technology, Utalii House, Uhuru Highway, P.O. Box 30568, Nairobi - 00100, Kenya; 3 Kenya Medical Research Institute, KEMRI/CDC Research and Collaboration programme, Kisumu-Maseno Rd, Kisian, P.O. Box 1578, Kisumu - 40100, Kenya; 4 Department of Clinical Epidemiology (KEBB) J1B.207-1, Academic Medical Centre, University of Amsterdam, P.O. Box 22660, 1100 DD Amsterdam, The Netherlands; 5 Global AIDS Programme, US Centres for Disease Control and Prevention, P.O. Box 54840, Nairobi - 00200, Kenya; 6 Global AIDS Programme, Center for Disease Control and Prevention, 1600 Clifton Road, NE, Atlanta, GA 30333, USA

**Keywords:** health-seeking, tuberculosis, diagnosis, delay, qualitative

## Abstract

**Background:**

The two issues mostly affecting the success of tuberculosis (TB) control programmes are delay in presentation and non-adherence to treatment. It is important to understand the factors that contribute to these issues, particularly in resource limited settings, where rates of tuberculosis are high. The objective of this study is to assess health-seeking behaviour and health care experiences among persons with pulmonary tuberculosis, and identify the reasons patients might not complete their treatment.

**Methods:**

We performed qualitative one-on-one in-depth interviews with pulmonary tuberculosis patients in nine health facilities in rural western Kenya. Thirty-one patients, 18 women and 13 men, participated in the study. All reside in an area of western Kenya with a Health and Demographic Surveillance System (HDSS). They had attended treatment for up to 4 weeks on scheduled TB clinic days in September and October 2005.

The nine sites all provide diagnostic and treatment services. Eight of the facilities were public (3 hospitals and 5 health centres) and one was a mission health centre.

**Results:**

Most patients initially self-treated with herbal remedies or drugs purchased from kiosks or pharmacies before seeking professional care. The reported time from initial symptoms to TB diagnosis ranged from 3 weeks to 9 years. Misinterpretation of early symptoms and financial constraints were the most common reasons reported for the delay.

We also explored potential reasons that patients might discontinue their treatment before completing it. Reasons included being unaware of the duration of TB treatment, stopping treatment once symptoms subsided, and lack of family support.

**Conclusions:**

This qualitative study highlighted important challenges to TB control in rural western Kenya, and provided useful information that was further validated in a quantitative study in the same area.

## Background

Although Kenya has a well-organized National Tuberculosis Control Programme, the rate of TB disease remains among the highest in the world. The country is ranked 13^th ^among the 22 TB high burden countries that constitute 80% of the global incidence of TB cases [1]. In 2006, there were 115, 234 new TB cases identified in Kenya, a case notification rate of 286/100,000 [2]. As in many African countries, these figures, although high, are still likely to reflect under-reporting of TB cases [3]. This makes the diagnosis and treatment of TB an important public health concern. Unless TB cases are identified and treated early and in greater numbers, TB-related morbidity, mortality, and drug resistance are expected to increase [4]. A great challenge to a tuberculosis control programme is ensuring that TB patients seek diagnosis in a timely manner and, once diagnosed, adhere to treatment.

In any cultural context, a precondition of health-seeking behaviour is the recognition and interpretation of symptoms by the individuals affected and by those around them [5,6]. Who is consulted once symptoms are recognized will depend on pre-existing beliefs about the likely meaning of the symptoms and the availability and accessibility of the various potential sources of help (traditional, spiritual, western medicine) [5]. This availability of multiple sources of care, combined with uncertainty about TB symptoms, stigma, and problems of access and affordability may further lead to considerable delays in diagnosis and treatment of TB. Being knowledgeable about "therapeutic narratives" *i.e*., participant commentaries on illness progression, health seeking options and related events, is important for gaining insight into the complex relationship of "traditional" and "modern" medical systems, which are useful for designing TB case-finding and health promotion activities. Most of these activities are aimed at reducing or removing barriers to timely presentation to appropriate health facilities so as to improve community health [7].

### Conceptual Framework

According to Kleinman [8], explanatory models represent specific personal and social meaning that persons assign to the experience of illness. They are usually derived from a person's knowledge, beliefs, and perception about health and illness and its causes, signs and symptoms, severity, transmission, options for treatment, and prognosis. The sources of a person's health beliefs may include ideas from the professional health sector (the person's exposure to the teaching of health care professionals); the popular sector (the social network in which beliefs and explanations about illness are shared by family and friends, and how they personally experience their symptoms, the ability to function, and stigma of illness); and the folk or traditional sector (beliefs originating from oral tradition or folk healers and non-professional specialists). Explanatory models are not static, and so a person may hold beliefs from each of these sectors. To gain an accurate picture of health seeking behavior, we must pay careful attention to the cultural sensitivity and appropriateness of data collection methods [9].

Despite their demonstrated negative effects on global TB control, little is known about the extent and duration of delay in seeking care and its determinants and default from TB treatment among patients in rural Kenya. One previous study in Kenya used participant observation and informal interview of individual patients to assess social and cultural factors of TB treatment default [10]. In another study, focus group discussions (FGDs) were used to assess community beliefs and knowledge about causation, transmission, symptomatology, and treatment-seeking behavioural patterns in hospitalized TB patients and suspects, as well as attitudes towards the disease and afflicted persons [11]. In the latter study, even though FGDs allow participants to build on points or disagree with each other, using FGDs may not be the best method for gathering sensitive information, as participants may be reluctant to share such information in the presence of others [9].

**Figure 1:**
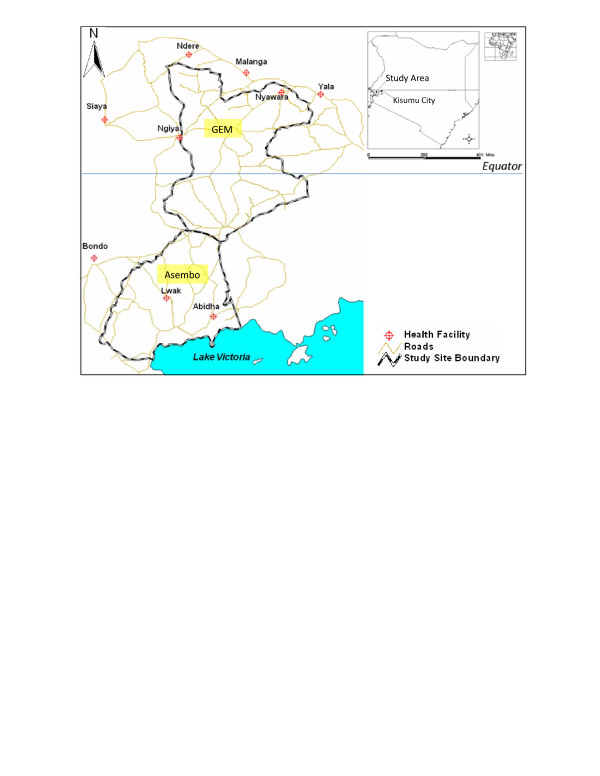
**The Map of the study site showing location of the study health facilities**.

To better understand the perception of TB illness in rural western Kenya [5], we explored culturally based explanations of illness and health among tuberculosis patients newly enrolled in the district TB treatment programme, and served in selected public health facilities in Asembo and Gem areas of Nyanza, western Kenya (Figure 1). Participants' own experiences were elicited through a free-response format using open ended questions [6]. In addition, this explored the knowledge, beliefs, and perceptions about TB signs and symptoms, severity, causation, transmissibility, treatment, and potential barriers to possible future treatment adherence, including the role of stigma as reported by study subjects.

## Methods

### Study sites and subject selection

This study was carried out in Asembo (0° -14' S and 34° 22' E) in Rarieda District and Yala and Wagai Divisions of Gem (0° 6' S and 34° 23' E) in Siaya District, Nyanza province, western Kenya (Figure 1). From 2002, The Kenya Medical Research Institute and Centres for Disease Control and Prevention (KEMRI/CDC) have been supporting a Health and Demographic Surveillance System (HDSS) in this area, the *"DSS area"*, and has been described in detail [12,13].

Kenya census for 2009 indicates that the population of Nyanza Province is 5,442,711 persons, with Rarieda and Siaya districts having populations of 137,755 and 550,224 respectively. The same census showed the populations for Asembo and Gem to be 64,509 and 83,059 people respectively. Nyanza province is very poor, with more than two-thirds of individuals living below the poverty line [14]. It has very high TB rates. For example, in 2006 there were 23,273 new TB cases reported (case notification rate of >400/100,000 population); 85% were of pulmonary TB [2]. Among TB cases aged 15-49 years, HIV prevalence was 73%, compared to 15% in the general adult population. Generally, the diagnosis of TB is made on the basis of sputum smear examinations or chest *X*-ray.

Study sites included 3 district hospitals of Bondo, Siaya, and Yala (all provide basic in-patient and out-patient curative and preventive health care services); five public and one mission health centres (all provide mainly ambulatory preventive and curative services) (Figure 1). The catchment areas for each of the facilities include parts of the HDSS area and the facilities serve low- to middle-income clients. The facilities were selected on an even geographical distribution within the study area and on the basis of their ability to provide acceptable TB treatment. They had a qualified staff in charge, and an adequate laboratory for diagnosis and follow-up. Each facility was visited daily Monday-Friday, between September 1 and October 31, 2005.

### Sample and recruitment

A convenience sample was used rather than random sampling because it has the advantage of identifying patients who are willing to actively participate. Thirty-one patients [18 women aged between 20 and 45, mean 28.3 (SD 7.1) years and 13 men aged between 22 and 52, mean 37.3 (SD 9.9) years], residing in an area of western Kenya with a Health and Demographic Surveillance System (HDSS) participated. They had attended treatment for up to 4 weeks on scheduled TB clinic days in September and October 2005.

Potential participants were newly diagnosed TB patients attending the study health facilities for no more than 4 weeks. They were systematically invited during weekdays during the study period to take part in an in-depth interview after their routine consultation. The purpose of the study was explained and informed consent was obtained and documented. Interviews were conducted by trained staff; anonymity was assured, with no names used throughout the study. Respondents were informed that participation would not affect their treatment. All chose to participate. To avoid interference with and from health workers, interviews were conducted in a private room.

### Interviews

All materials for the study, including the informed consent form and the interview guide, were developed in English and written at or below a fourth-grade reading level. The documents were then translated into Dholuo (the local language for all the participants). The Dholuo version was then independently translated back into English. The results of the translated English version were compared with the original English version and decentered, *i.e*., both the source and the target language versions were modified to make them congruent [15,16].

Interviewers used open-ended discussion guides and covered areas such as symptom onset, perceived cause, treatment seeking trajectory, disclosure, and time to seeking care. Questions also explored experience of stigma, whether enacted (actual experiences of stigma) or felt (anticipated stigma) [17], relating HIV and TB and potential reasons for possible future non-adherence to treatment. The questions were repeated, with the interviewer probing for detail until the patient's story of enrolling in TB treatment was completed. Interviews continued until all categories were well defined and saturated; no new or relevant data emerged after interviewing thirty-one participants [18]. Interviews generally lasted between 60 and 90 minutes, and were audiotaped with the participant's permission, transcribed, and translated into English.

### Ethical clearance

The study protocol was approved by the Ethical Review Committee at the Kenya Medical Research Institute (KEMRI) and the Centres for Disease Control and Prevention (CDC), Atlanta, Georgia, USA.

### Data analysis

Data analysis was performed using Glaser and Strauss's (1967) grounded theory method, which involves continuous and simultaneous data collection, coding, and subsequently grouping into categories to produce "themes" [19]. Although the purpose of this study was not theory development, we felt that the grounded theory approach would be the most effective method of analysis.

## Results

### Symptoms and perceived causes

Respondents' stories started with a variety of non-specific symptoms, such as fever, headache, chest pain, fatigue, and body pain. Persistent and prolonged cough was the TB symptom most frequently mentioned by most participants. Participants' awareness of TB, its symptoms, and the seriousness of the disease was poor. When asked about the cause of their symptoms, 14 responded "I don't know" (i.e., had no knowledge at all), while 8 had some knowledge or misperception about the cause of their symptoms. Some participants thought that environmental factors, such as inhaling smoke and hot air from charcoal burning or sharing a house with domestic animals, were the cause of their symptoms. Other patients thought that TB is acquired from alcohol, water, or sharing utensils:

"*I got TB from cold drinks... drinking stagnant water... sharing food or utensils*" (Interview summary 19, man, age 34)

Other perceived causes of their symptoms were predominantly related to physical factors, including chest injury or hard physical work:

"*TB got me because of the hard work that I do [riding 'boda-boda' (bicycle) taxi]*" (Interview summary 30, man, age 24)

A number of participants also perceived TB to be inherited:

"*My mother told me that I got it since it is in our lineage [inherited]*" (Interview summary 13, woman, age 24)

Others perceived it to be caused by spiritual or evil forces (7), often blaming neighbours for putting a curse on them:

"..... *some claimed that it [my sickness] is "chira" [i.e., curse/bad omen] type of cough or ... somebody was trying to bewitch me ... we requested a religious healer to pray for me" *(Interview summary 11, woman, age 30)

### Delay in seeking health care

Although all participants reported symptoms typically associated with TB, many delayed seeking treatment. The period from symptom to diagnosis varied considerably both in length (3 weeks to 9 years) and in number of efforts to seek treatment [median 2, IQR (1, 3)]. Various reasons for delaying treatment are summarized in Table 1. Seventeen participants stated that they delayed seeking care because they were "not concerned" about their symptoms:

**Table 1:** Reasons for delay in seeking health care in Asembo and Gem areas of Nyanza, September and October 2005

Reason for delay	(Number of participants)*
Thought symptom not serious/normal cough	(17)
Lack of money	(9)
Not encouraged by family	(2)
Health provider refused me care and asked me to go to another facility	(1)
There was nobody to leave my baby with	(1)
Didn't have time to go	(1)
Don't like long queues at health facility	(1)
Don't like ways the nurses talk to patients	(1)
There was nobody to relieve me at my place of work	(1)
I was alcoholic	(1)
	

Numbers in parentheses show the number of patients attributed to the reason. They do not add to 31 as some patients gave more than one reason.

"*I thought that it was a normal cough" (*Interview summary 2, woman, age 30)

**Figure 2:**
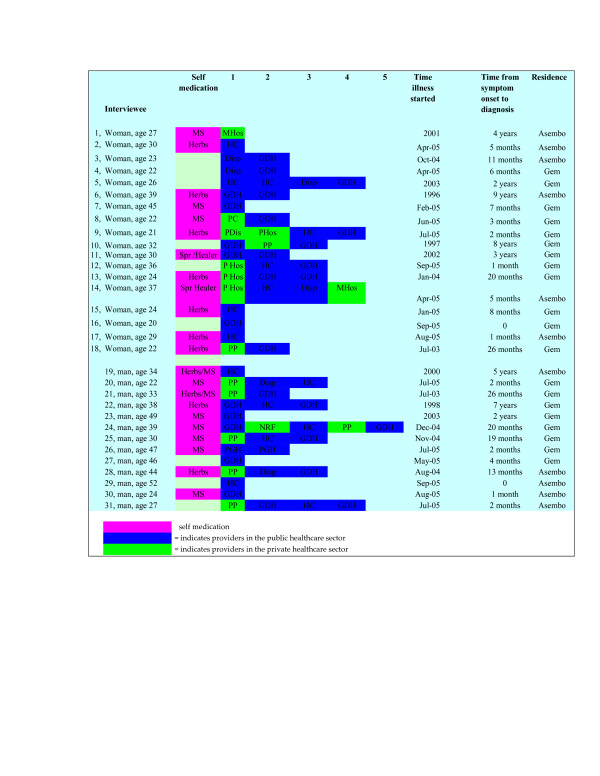
**Attempts made by TB patients newly enrolled in the district tuberculosis treatment programme, Nyanza, western Kenya, September and October 2005**.

Prolonged self-treatment prior to consulting a biomedical health facility was widespread among the participants (22) (Figure 2). Often patients chose self-treatment, for example using herbs or buying medication from kiosks/pharmacies. Those who attributed their TB to a curse or witchcraft contacted spiritual healers.

More than half of the patients (17) sought advice of a close relative, and most delayed seeking professional care because they were advised to seek help from herbal/spiritual healers (Figure 2):

"...*when my condition had worsened... my mother took me to some person.... who boiled some herbs for me to drink*...." (Interview summary 18, woman, age 22)

Preference for a care provider whom a patient knew and trusted along with economic constraints also led to considerable time elapsing before obtaining a diagnosis and appropriate treatment, as narrated by one patient:

"...*I just decided much earlier on my own to go to "Russia" (in reference to Nyanza Provincial General hospital, a Provincial referral public health facility in the study region, built by Russian government as an aid to the Kenyan government) but not Yala that is nearby because I knew some of the doctors there (Nyanza Provincial General) ... but I could not do it early enough due to lack of money*" (Interview summary 26, man, age 47)

Another patient who took 26 months to be diagnosed said:

*"---- I was told to go for an X-ray test at Yala ...due to lack of money, I stayed for a while before going for the test ..." *(Interview summary 18, woman, age 22)

Other causes of delay included inconveniences with work or dislike of long queues at the health facility:

"*At my place of work I am alone.... I could not go to hospital, so I used to buy drugs to reduce the pain*" (Interview summary 24, man, age 39)

"*I was avoiding queuing at the hospital*......" (Interview summary 23, man, age 49)

### Decision to seek medical attention

When symptoms persisted or returned after the initial treatment attempt or the respondents perceived the illness as being serious, the patient was motivated to visit a health facility in an attempt to receive proper diagnosis.

Figure 2 shows the detailed efforts to seek help by study participants until the time of enrollment in the district tuberculosis treatment programme. Overall, 19 patients first visited a care provider in the public health sector, where half of them (10) were diagnosed with TB. Twelve first visited providers in the private health sector, where only one was diagnosed with TB. On the second attempt to seek treatment, only 8 respondents who went to a public health facility were diagnosed as having TB. Of the remaining 12 patients, seven were diagnosed as having TB on their third attempt, and five on their fourth or fifth attempt, of which one of these was in a private facility. Thus, in only two patients (the first and fourteenth), a diagnosis was made at a private (mission) facility. The rest were diagnosed at public facilities

### Health provider delay of TB diagnosis

Nine of the 19 respondents who visited public sector health care providers and 11 of 12 who visited private sector providers reported that health care givers failed to consider TB early in the course of their illness (Figure 2). This resulted in unnecessary trips to the health facilities, delayed diagnosis and care, and inefficient use of scarce financial resources. A patient who took 20 months to be enrolled in the treatment programme reported:

"*I went to Yala sub-District hospital... I was told I had malaria... then Kenyatta National hospital...told had malaria...then to Malanga Health centre... was told had malaria ...then to a private hospital... I was given eleven injections...to Yala again told to go for X-ray and VCT... then was found to have TB*" (Interview summary 24, man, age 39)

### Social stigma associated with TB

The family was perceived to be influential in helping patients seek care, through economic support and in assisting to identify appropriate health facilities for medication. When asked: *"Did you tell your people at home that you have TB?" *30 respondents stated that their relatives and friends knew that they had TB, and that they themselves had told them. When asked: "*Do they treat you differently in any way since you have TB?*" to explore *enacted *(actual experiences of) stigma, majority (24) stated that they experienced no significant change in the behaviour of people towards them, and maintained that no precautions against them were taken by other members of the family. Social interactions continued as normal and were supportive in assisting them in their treatment:

"**... ***they make sure that I eat... and I take my drugs" *(Interview summary 20, man, age 22)

However, the remaining six confirmed that there were negative reactions when they learned they had TB, as family members tried to avoid close contact:

"*They have given me separate set of utensils and I don't share meals*..." (Interview summary 26, man, age 47). Another patient stated: "*They treated me differently as if I had HIV because of my cough and mass wasting*" (Interview summary 10, woman, age 32)

This revealed existence of some enacted social stigma associated with TB in the study area.

### HIV "cross-stigmatization"of TB

About half (15) of the participants felt that there is an association between TB and HIV/AIDS. When participants were asked: "If people with TB are asked to take an HIV test, how would it affect their going for a TB test?" One patient narrated:

"*---- I have TB and hear from people that I will be tested for HIV, and that can make one not go to be tested for TB ---- if you hear they will test that (HIV), then you decide just to continue coughing instead*" (Interview summary 12, woman, age 36)

Respondents were also asked to state whether they were uncomfortable talking to certain people about their TB problem. This was aimed at exploring anticipated stigma among the participants. Eleven (11) participants had felt stigma, as summarized here by a participant:

"... *I was uncomfortable because I was thin.... people here associate it (wasting) with AIDS..." *(Interview summary 20, man, age 22)

## Potential reasons for defaulting on treatment

Patients must complete the TB treatment regimen to be cured and to prevent transmission and development of drug-resistance. Reasons for defaulting of treatment are varied and are also summarized in Table 2. The most common reasons included limited communication with providers and lack of their involvement in the treatment process. When asked how long treatment would take, 14 patients lacked knowledge on the duration of treatment:

**Table 2:** Potential reasons for defaulting on treatment in Asembo and Gem areas of Nyanza, September and October 2005

Reason given	(Number of participants)*
Lack of knowledge/misperception about duration of TB treatment	(14)
When I start feeling well	(4)
If my disease worsens	(4)
Alcoholism	(2)
Laziness	(2)
Medicine stockouts	(2)
Delays in being served at health facility	(2)
Too-long period of medication	(1)
There is nobody to get me medication	(1)
If I am discouraged by people	(1)
Side effects like vomiting	(1)
No transport	(1)
Stress from family	(1)
Smoking	(1)
If ever I interrupt my treatment	(1)

Numbers in parentheses show the number of patients attributed to the reason. They do not add to 31 as some patients gave more than one reason.

"....*I was just started on the medication... I haven't been told (duration of treatment)*" (Interview summary 8, woman, age 22)

Patients may fail to complete their treatment if they feel the treatment lasts too long, particularly after symptoms disappear. Accounts indicate that patients stop taking their medication because they feel better as a result of the early stages (intensive phase) of treatment. This was associated with a poor understanding of the need to continue treatment even after the symptoms of illness have subsided:

"...*I don't usually finish taking my drugs... If I start taking these drugs and feel well, I will leave but so long as I still cough, I will just continue*" (Interview summary 3, woman, age 23)

In some cases perceived side effects resulting from chronic hunger (6) could lead to defaulting treatment:

"...*these drugs make one feel fatigue, improved appetite...when there is no food, it is not easy*..." (Interview summary 27, man, age 46)

Anticipated lack of adequate supplies of TB drugs at health facilities, stated by eight of the respondents, was a potential contributor to potential poor compliance with treatment:

"*I will stop medication if at all I come to collect drugs from the dispensary and I find that there are no drugs..*." (Interview summary 19, man, age 34)

The social support of family members is also important, especially during the intensive phase of treatment. A male patient explained how lack of family support can affect adherence:

"*Stress from family members can discourage one from taking drugs*" (Interview summary 24, man, age 39)

## Discussion

In general, most participants had low knowledge of TB. A majority self-treated before seeking care from the formal health sector, and most sought care at public health facilities. A diagnosis of TB was confirmed at public facilities in all but two of the patients. However, only half of patients visiting these public facilities and one of the twelve patients who visited private facilities for the first time were diagnosed with TB.

### Understanding of TB aetiology

Many TB patients started with relatively non-specific symptoms, partially explaining the observed delay in seeking care or difficulties in obtaining appropriate treatment among the study participants visiting health facilities. Many participants also held ideas about TB, most of which were inconsistent with the fact that bacteria cause TB. Client explanations of aetiology of TB in this study suggest the presence of popular and folk beliefs [20]. Some patients mentioned sharing utensils, food, and water as the cause of their TB; others attributed TB to hard physical work or exposure to cold or smoke. Still others believed that TB is inherited. Similar findings have been reported in a previous study [11] and elsewhere [21]. These findings are of concern, since participants being newly enrolled in a district treatment programme should have been clearly informed about the aetiology of TB at the start of their treatment.

### Health care seeking

Early detection and treatment of tuberculosis is critical to controlling the disease [22]. Although cough and fever were common symptoms at onset, these symptoms alone did not always prompt patients to seek medical treatment early. Symptoms in the early stages of TB are not very specific and may be attributed to self-limiting illnesses, such as viral infections, and only when symptoms become worse or persist will the person consult a health service. Symptom misinterpretation has been associated with patient delay in other studies internationally, where they are attributed to external causes such as overwork or exposure to cold [23,24]. This delay in health-seeking behaviour is likely to have increased the risk of morbidity, mortality, and transmission of TB to contacts.

Personal and community knowledge of TB and interpretation of health beliefs influences attitudes and health-seeking behaviour significantly [25-27]. Self-treatment, involving a variety of home remedies and traditional and modern drugs, is the first step in the health-seeking behaviour process. This observation is consistent with previous studies, and is linked with the perception of the seriousness of the symptoms and the label the patient attaches to his/her condition [9,11,28]. Persons with untreated sputum smear positive TB can infect 10 to 14 others in a year [29]. There is a need for interventions that encourage symptomatic individuals to seek medical help early.

### Missed diagnoses at health facilities

Lack of money for diagnostic tests and low suspicion of TB at health facilities caused further delay in obtaining correct treatment, once a person decided to seek care at a health facility. Only half of patients who visited public health facilities and only one among those who visited a private facility for the first time were diagnosed with TB. Though we did not investigate this, an extensive review of previous studies show that most private hospitals serving the urban and rural poor in the developing world are ill-equipped and their staff unqualified, hence the low suspicion for TB among patients who visited these facilities [30]. This confirms findings from a previous study in Kenya that showed that health units failed to investigate chronic coughs in a certain proportion of TB suspects [31]. Even though a good proportion of patients visited providers in the private sector, there was a marked decrease in the number of patients seeking care at these facilities. For instance, at attempt 1, 12 out of the 31 sought diagnosis at a private facility; attempt 2, only 3 out of 31 went to a private facility; attempt 3, zero; and attempt 4, only 2 (Figure 2). While this may suggest a loss of confidence in these facilities by patients, more investigation is needed to explain this shift in health care seeking.

There are many consequences of missing the diagnosis of tuberculosis, and this raises several programme and policy issues [32]. For the patient, misdiagnosis and faulty treatment leads to loss of scarce time and money in the search for treatment, and may increase the duration of illness and the possibility of death. For public health officials, misdiagnosis causes an underestimate in the rate of incident TB, and increases the duration of infectivity. Interventions that could improve the likelihood of TB diagnosis at health facilities may include implementation of standard screening procedures, additional training of health care workers, education of patients (so that they expect and request diagnostic testing for TB when appropriate), and better access to and reduced costs for diagnostic tests.

### Stigma

Very few people felt that their having TB had affected their relationships with friends and family, and if it did, it appeared to elicit more support. This finding is consistent with other studies that have documented social stigma of TB to be more extra-familial than intra-familial [33]. This is important because social support provided by family often plays a pivotal role in promoting early TB diagnosis and adherence to treatment [11,34]. However, a small proportion of our participants expressed how they were treated as though they had HIV due to their cough and mass wasting.

The association between HIV and TB could extend existing TB stigma, as observed elsewhere [35,36]. Our study also suggests that stigma related to HIV infection may reduce TB test uptake among TB suspects. Stigma is linked to concealment of symptoms, treatment default, isolation from support networks, decreased self-esteem, self-perception, and self-care [37]. Health education should therefore aim at reducing tuberculosis-related stigma. The key here is the Provider Initiated Testing and Counseling programme, which is now part of the integrated service delivery in Kenya. This programme promotes the awareness that TB is not always associated with HIV [38], and that it can be cured in both persons with and without HIV.

### Potential reasons for defaulting on treatment

One of the objectives of our study was to assess the potential reasons for defaulting on treatment. We found that our study participants could decide to interrupt TB treatment because of lack of knowledge on the duration and the importance of completing the full treatment course, improvement in symptoms, drug stock-outs at the health facility, side effects of TB medications, and lack of social support. These findings are consistent with other studies [39,40]. Patient narratives seemed to suggest lack of communication and lack of patient involvement in the treatment process, leaving them poorly equipped to take an active role in managing their own health. They are, therefore, poorly prepared to make informed decisions about their treatment [41]. These are factors that have been found to be associated with high rates of treatment default [42]. An improvement in these aspects of TB treatment is crucial in encouraging patients to continue with treatment for the full duration of the regimen.

### Limitations

This was a sociological study involving in-depth interviews. For this reason, the sample size of our investigations was small, with only 31 respondents selected by purposive sampling method which could have introduced bias into the results. We tried to reduce recall bias by including only patients who were diagnosed no more than four weeks before the interview. Undetected "cases" were not included, and have been addressed in our recent study that actively identified TB cases in a TB prevalence survey in the same study area (AH van't Hoog, BJ Marston, JG Ayisi, JA Agaya, O Muhenje, LO Odeny, J Hongo, KF Laserson, MW Borgdorff: Risk Factors for TB Case Finding in a high-HIV population: a Comparison of Prevalent and Self-reported TB Cases, submitted). Our outcome measure of delay in seeking care was self-reported, and no attempt was made to verify the patient reports. We did not collect HIV status of the interviewed participants, hence a possibility of misclassification of non-TB symptoms as those of TB, with consequent unrealistically long periods of delay (several years). Our findings may only be applied to the factors studied, for this study did not intend to assess other important aspects of TB control, such as perceptions and attitudes of health care providers, the community, and suspects, nor did it assess the quality of the TB control programme.

## Conclusions

Overall, the high number of patients who shared their TB diagnosis with family members and the subsequent high numbers who did not experience enacted stigma in this study population are very encouraging for the district TB control programme. However, although we explored the experiences of only a small number of individuals, several recurring themes highlight the challenges for TB control in rural western Kenya. Lack of awareness of treatment duration, stock-outs, and lack of family support were reported as potential reasons for treatment default.

The low knowledge of TB among the participants and low suspicion of TB among health facility staff, together with few reported experiences of both enacted and felt HIV and/or TB-associated stigma, should be of concern to TB control programme in the study area. In addition, the fact that only two patients of 12 consulting the private sector were diagnosed confirms a lack of awareness and engagement of the private sector with TB control. This calls for strengthening the collaboration between private health facilities and the Division of Leprosy, Tuberculosis and Lung Disease in Kenya.

## Abbreviations

Herbs: Herbalist; Disp: government dispensary; MS: medical shop/drug retailer; PDis: private dispensary; Trad Pr: traditional practitioner; GDH: government district hospital; PHos: private hospital; MHos: mission hospital; PGH: government provincial general hospital; PP: private practitioner; NRF: None governmental run facility; HC: governmental health centre; Spr: spiritual healer.

## Competing interests

The authors declare that they have no competing interests.

## Authors' contributions

JGA, AHvH, BM conceived of the study, and participated in its coordination.

All authors participated in the design. JGA, JAA and AHvH carried out the study. WM, PON and OM assisted in the analysis and data interpretation. JGA and AHvH drafted the manuscript. All authors read and approved the final manuscript and confirm that the content has not been published elsewhere and does not overlap or duplicate their published work.

## Pre-publication history

The pre-publication history for this paper can be accessed here:

http://www.biomedcentral.com/1471-2458/11/515/prepub
